# Sub-Poissonian photon statistics in quantum dot-metal nanoparticles hybrid system with gain media

**DOI:** 10.1038/s41598-019-46576-z

**Published:** 2019-07-12

**Authors:** Yujing Wang, Han Ye, Zhongyuan Yu, Yumin Liu, Wenbin Xu

**Affiliations:** 1 State Key Laboratory of Information Photonics and Optical Communications, Beijing University of Posts and Telecommunications, Beijing, 100876 China; 2 Science and Technology on Optical Radiation Laboratory, Beijing, 100854 China

**Keywords:** Nanocavities, Quantum optics

## Abstract

In this paper, we theoretically demonstrate the sub-Poissonian photon statistics in gain-assisted quantum dot-metal nanoparticles (QD-MNPs) hybrid system with nanoscale footprint. The gain media is introduced to reduce the dissipation of localized surface plasmons and consequently the quality factor of MNPs is improved by adjusting the gain coefficient. Simulated by finite element method (FEM), the Fano resonance is observed in the absorption cross section spectrum of the hybrid system. Considering MNPs and gain media together as a single mode cavity, the system is investigated within the framework of cavity quantum electrodynamics by fitting necessary parameters with FEM. The numerical results show that the coupling between QD and MNPs falls in strong coupling regime and zero delay second-order autocorrelation function *g*^2^(0) = 0.356 can be achieved with proper choice of gain coefficient. Moreover, the sub-Poissonian photon statistics can be maintained in a large variation range of gain coefficient and a certain degree of detuning between QD and cavity is allowed.

## Introduction

Solid-state single photon source (SPS) is a key component in the quantum computation^1^ and quantum communication systems^2^. The photon-counting statistic of single photon is sub-Poissonian, which is the manifestation of non-classical state^3^. As one major category of building blocks to realize the generation of sub-Poissonian light, the integrated quantum dot (QD) – microcavity coupling system attracts huge research interest in the last two decades. Microcavities, including micropillar cavity^4–6^, photonic-crystal cavity^7–9^, nanobeam cavity^10,11^, and microdisk cavity^12,13^ etc., are of great help for confining the light field, thus coupling QD with microcavities can efficiently enhance the interaction between light and matter. Both of the quality factor (Q factor) and effective mode volume play crucial roles on the performances in these schemes. Moreover, reducing the footprint of system from micrometer to nanometer is still under pursuit. The localized surface plasmons (LSPs) have been observed not only enhance the intensity of light field, but also possess an extremely small mode volume below the diffraction limit^14^. Recently, a promising nanoscale scheme based on QD-LSPs coupling system has been proposed for the generation of single photon^15^.

As a prototype of structures for LSPs, the metal nanoparticles (MNPs)^16–18^ with various shapes have been demonstrated, including sphere^19,21^, ellipsoid^20,22^, channel type^23^ and antenna type^24,25^. When placing a QD near the MNPs, Fano resonance was theoretically obtained in both spherical^19^ and double ellipsoidal nanoparticles^20^, reflecting the interaction between QD and MNPs. In experiments, Hartsfield *et al*.^21^ successfully demonstrated the QD induced anisotropy to MNP and the modification of scattering spectrum of spherical MNP by manipulating the QD position. When QD-double metal ellipsoids system was excited by femtosecond laser pulses, the Fano resonance could undergo an ultrafast reversal^22^. In QD-bowtie silver nanoparticles coupling structure, a transparency dip in the scattering spectra of system was experimentally observed, and the coupling rate reached 120 meV. As a manifestation of strong coupling, the above phenomenon can be described as the vacuum Rabi splitting^25^. Furthermore, several schemes realizing the generation of sub-Poissonian light have been proposed in recent years^15^. Straubel *et al*.^26^ proposed adiabatic elimination theory to describe quantum optical evolution and demonstrated the generation of sub-Poissonian light in quantum emitter-plasmonic nanoantenna hybrid system based on this theory. In quantum emitter-channel plasmons system^23^, the value of zero delay second-order autocorrelation function *g*^2^(0) was observed less than 0.5 in experiment. Stronger coupling is beneficial to further reduce *g*^2^(0)^27^, however, it is limited by the high attenuation rate of LSPs. Fortunately, the dynamic feedback between LSPs and gain media was demonstrated providing possibility to make up this loss by photon-plasmon coupling. If carefully adjusting the gain coefficient, the dissipation of cavity can be efficiently reduced^28,29^.

In previous studies, Wu *et al*.^20^, Shah *et al*.^22^ and Hensen *et al*.^24^ have fitted the classical field and quantum field to extract the important quantum parameters (e.g. coupling strength *g* and decay rate *κ*) in QD-LSPs coupling systems. However, the photon statistical properties were not further explored. In contrast, Koenderink^15^ and Bermúdezureña *et al*.^23^ only measured *g*^2^(0) directly in experiments without theoretical estimations in advance. In this work, a predictive framework is demonstrated to theoretically evaluate the photon statistical properties in the QD -MNPs coupling system. Considering MNPs and gain media together as an equivalent cavity, we calculate the zero delay second-order correlation function *g*^2^(0) and intra-cavity photon number *N* using cavity quantum electrodynamics (cQED)^30^, based on the quantum parameters fitted from the absorption cross section spectra simulated by finite element method (FEM). This process provides an approach to design coupling system with desired performances. More importantly, in this paper we explore the sub-Poissonian photon statistics in the gain-assisted QD-double gold semi-ellipsoids hybrid system.

## Structure and Theory

**Figure 1:**
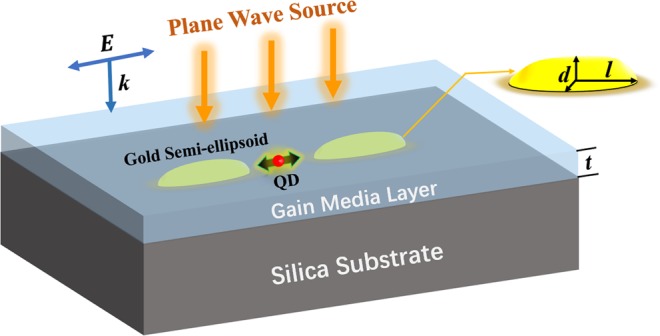
Illustration of Ag_2_S QD- gold semi-ellipsoids hybrid system.

As illustrated in Fig. 1, the nanoscale hybrid system simply consists of one pair of gold semi-ellipsoids, one QD and one layer of gain media covering them. The semi-major axis *l* and two semi-minor axes *d* of semi-ellipsoids are 50 nm and 10 nm, respectively. Two MNPs are aligned along the major axes with a gap of 4 nm. Considering the stability of structures sitting on substrate, the semi-ellipsoids are more appropriate than the ellipsoid dimer used in refs^20,22^, and they could likely be fabricated using the state-of-the-art electron-beam lithography and template-stripping methods^31,32^. In FEM, the dielectric constant of gold semi-ellipsoids is described by Drude model, which is commonly used when simulating the optical response of plasmonic resonant MNPs^33^ and has the function of

1 $${{\epsilon }}_{s}(\omega )={\varepsilon }_{\infty }-\frac{{\omega }_{p}^{2}}{{\omega }^{2}+i{\rm{\Gamma }}\omega },$$

where *ε*_∞_ represents the material dielectric constant at high frequency, *ω*_*p*_ is the plasma frequency, and Γ is the damping constant. As in ref.^33^, we take *ε*_∞_ = 9.5, *ℏω*_*p*_ =  8.96 eV and *ℏ*Γ = 69 meV. The spherical Ag_2_S QD with 3.4 nm diameter, which emits photons near 950 nm^34^, is positioned at the center of the gap. The QD here is treated as a dielectric particle and all higher-order transitions are ignored^20^. The dielectric constant of QD is modeled as Lorentzian function^20,22^

2 $${{\epsilon }}_{q}(\omega )={{\epsilon }}_{\infty }^{{^{\prime} }}-f\frac{{\omega }_{c}^{2}}{{\omega }^{2}-{\omega }_{c}^{2}+i{\gamma }_{q}\omega },$$

where ${{\epsilon }}_{\infty }^{{^{\prime} }}=4.84$ is the high-frequency dielectric constant of bulk Ag_2_S, *f* is the oscillator strength of the transition, and *γ*_*q*_ is the linewidth of QD. We take *f* = 0.1 and *γ*_*q*_ = 2 *meV*^20,22^. *ω*_*c*_ is the center frequency chosen to match the plasmon frequency of the MNPs. The Ag_2_S QD can be synthesized by one-step method reported in ref.^34^, in which the growth time and temperature play crucial roles on the size of QD and can be adjusted to make sure *ω*_*c*_ is suitable for the system. The precise positioning QD into the gap can be achieved by guided colloidal deposition^35^. Both MNPs and QD are placed on the silica substrate with typical refractive index 1.5^36^, which is widely used not only in simulations^20^ but also in experiments^23,25^. The covering gain media layer with thickness *t* = 20 nm is assumed made of doped silica, whose dielectric constant is (1.5 − *ik*)^2^, where *k* is the gain coefficient describing the interaction strength between the incident light and gain media^28^. The hybrid system is illuminated by a plane wave polarizing along the major axes of MNPs, which has been verified to be able to excite the bright mode of LSPs and was previously adopted to drive QD-MNPs coupling systems^20,22^. The field distribution and absorption cross section spectra are numerically calculated by FEM. The thicknesses as well as the lengths of air and substrate are set as 950 nm, which is close to one wavelength. This size is verified large enough for consistent optical response of the structure. Moreover, perfect matched layers (PML) with 475 nm thickness are built as the outer boundary.

Under the framework of cQED, the QD-MNPs hybrid structure is considered as a two-level QD-single mode cavity system^22^, which can be described by Jaynes-Cummings (JC) model^37–40^. The quantum evolution of the hybrid system is described by the master equation

3 $$\dot{\rho }=-i[ {\mathcal H} ,\rho ]+ {\mathcal L} (\rho ),$$

where ${\mathcal H}$ is the Hamiltonian, *L*(*ρ*) is the Lindblad superoperator representing dissipation and dephasing, and *ρ* is the density matrix. In more detail,

4 $$ {\mathcal H} ={\omega }_{s}{a}^{\dagger }a+{\omega }_{q}{\sigma }^{\dagger }\sigma -g({a}^{\dagger }\sigma +a{\sigma }^{\dagger })-E(t)\mu ,$$

where $E(t)=Ecos({\omega }_{l}t)=E/2({e}^{i{\omega }_{l}t}+{e}^{-i{\omega }_{l}t})$ is the driving field intensity, $({\sigma }^{\dagger },\sigma )$ is the raising and lowing operator pair for QD, $({a}^{\dagger },a)$ is the generation and annihilation operator pair for intra-cavity photons, *ω*_*s*_ and *ω*_*q*_ are the eigen-frequencies of cavity and QD respectively, and *g* is the coupling strength between them. *μ* = *μ*_*s*_ + *μ*_*q*_ is the total dipole operator consisting of ${\mu }_{s}={d}_{s}({a}^{\dagger }+a)$ and ${\mu }_{q}={d}_{q}({\sigma }^{\dagger }+\sigma )$, where *d*_*s*_ and *d*_*q*_ are the transition dipole moments of cavity and QD, respectively. Taking the rotating frame into consideration (at the driving field frequency *ω*_*l*_), formula (4) turns into

5 $$ {\mathcal H} ={\rm{\Delta }}{\omega }_{s}{a}^{\dagger }a+{\rm{\Delta }}{\omega }_{q}{\sigma }^{\dagger }\sigma -g({a}^{\dagger }\sigma +a{\sigma }^{\dagger })-E\mu ,$$

where Δ*ω*_*s*_ = *ω*_*s*_ − *ω*_*l*_ and Δ*ω*_*q*_ = *ω*_*q*_ − *ω*_*l*_. Another operator in master equation ${\mathcal L} (\rho )$ is given by

6 $$ {\mathcal L} (\rho )=\kappa  {\mathcal L} (a)\rho +{\gamma }_{1} {\mathcal L} (\sigma )\rho +2{\gamma }_{2} {\mathcal L} ({\sigma }^{\dagger }\sigma )\,\rho ,$$

with a form of ${\mathcal L} (x)\rho =x\rho {x}^{\dagger }-\frac{1}{2}{x}^{\dagger }x\rho -\frac{1}{2}\rho {x}^{\dagger }x$. Here, *κ* is the decay rate for cavity mode, and *γ*_1_ and *γ*_2_ are the spontaneous emission rate and dephasing for QD, respectively. If redundant decoherent photons induced by the gain media stay in the cavity, item $\kappa ^{\prime}  {\mathcal L} ({a}^{\dagger })\rho$ representing the decoherence channel should be added in Eq. (6), which is ignored in present work due to the assumption that nearly all decoherent photons generated by gain media are consumed in the photon-plasmon coupling. The absorption cross section spectra have been demonstrated as a bridge with good consistency between discrete dipole approximation, semi-classical model and cQED, when treating the coupling between QD and MNPs^22^. Following this idea, we determine the eight unknown parameters in Hamiltonian and Lindblad superoperator by fitting the spectra obtained from cQED and FEM. In order to find the stationary solution, we solve Eq. (3) for the steady-state matrix density *ρ*(*ω*). On the basis of *ρ*(*ω*), the quantum mechanical dipole is described as *μ*_*ω*_ = *T*_*r*_[*ρ*(*ω*)*μ*]^22^. The absorption cross section is *σ*_*abs*_(*ω*) = *P*_*abs*_(*ω*)/*S*_0_^20^, where *P*_*abs*_(*ω*) = (*ω*/2)Im[*μ*_*ω*_E^*^] is the average power absorbed by the dipole, and ${S}_{0}=\sqrt{{\varepsilon }_{d}}c{{\epsilon }}_{0}{E}^{2}/2$ is the incident flux. The complex polarizability is $\alpha (\omega )={\mu }_{\omega }/({{\epsilon }}_{d}E)$, thus the absorption cross section is written as

7 $${\sigma }_{abs}(\omega )=({k}_{d}/{\varepsilon }_{0}){\rm{Im}}[\alpha (\omega )],$$

where ${k}_{d}=\sqrt{{\varepsilon }_{d}}\omega /c$, and *ε*_*d*_ is the dielectric constant of the medium around the dipole. In FEM, *P*_*abs*_(*ω*) is directly obtained from the integral of power loss density

8 $${P}_{abs}(\omega )=1/2\iiint (\overrightarrow{J}\cdot \overrightarrow{E})dv,$$

where $\overrightarrow{J}$ and $\overrightarrow{E}$ are current density and electric field intensity, respectively. Using the above two methods, three pairs of *σ*_*abs*_ curves are determined, including isolated cavity, isolated QD and hybrid system. By fitting each pair of curves, we can finally determine all eight necessary quantum parameters^22^. Then, zero delay second-order correlation function ${g}^{2}(0)= < {a}^{\dagger }{a}^{\dagger }aa > / < {a}^{\dagger }a{ > }^{2}$ and intra-cavity photon number $N= < \,{a}^{\dagger }a >$ of the hybrid system can be evaluated under the framework of cQED. The thermal noises induced by gain media are not taken into consideration under cold reservoir limit in present work for simplicity^30^.

## Results and Discussion

**Figure 2:**
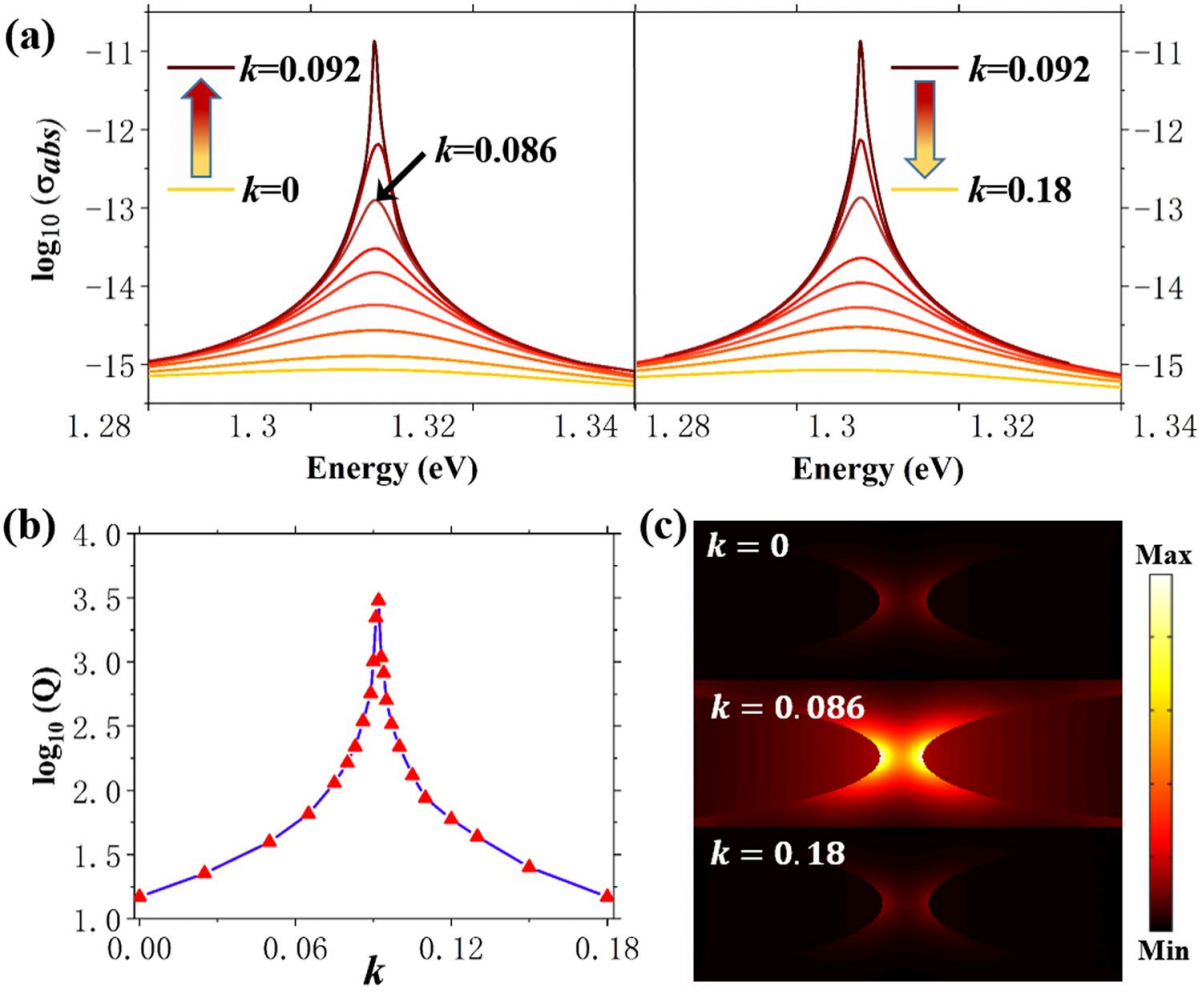
(**a**) FEM calculated absorption cross section spectra for the MNPs with different gain coefficient *k*. (**b**) Q factor of the MNPs as a function of *k*. (**c**) Normalized electric field intensity distribution around the MNPs with different *k*.

First, the impact of gain media on the optical properties of only MNPs are explored. As illustrated in Fig. 2(a), the full width at half maximum (FWHM) of absorption cross section spectrum clearly decreases first along with the increase of gain coefficient and reaches the minimum around *k* = 0.092, then broadening of spectrum can be observed with the further increase of *k*. The corresponding cavity quality factor *Q* is as low as 15 when gain media is absent or gain is too strong. By contrast, the maximum *Q* = 3006 appears around *k* = 0.092, indicating the dissipation of MNPs is efficiently limited. This resonance-like enhancement of quality factor and absorption cross section can be attributed to the dynamic feedback between MNPs and gain media, which can make up loss by photon-plasmon coupling^28,29^. As shown in Fig. 2(c), compared with *k* = 0 and *k* = 0.18, the normalized intensity of electric field near MNPs is far stronger at *k* = 0.086, indicating that gain media helps to enhance the field intensity when *k* is properly chosen. Since the energy of incident wave ℏω (wavelength) ranges from 1.28 eV (970 nm) to 1.34 eV (927 nm), *k* is assumed constant in such narrow wavelength range in present work.

**Figure 3:**
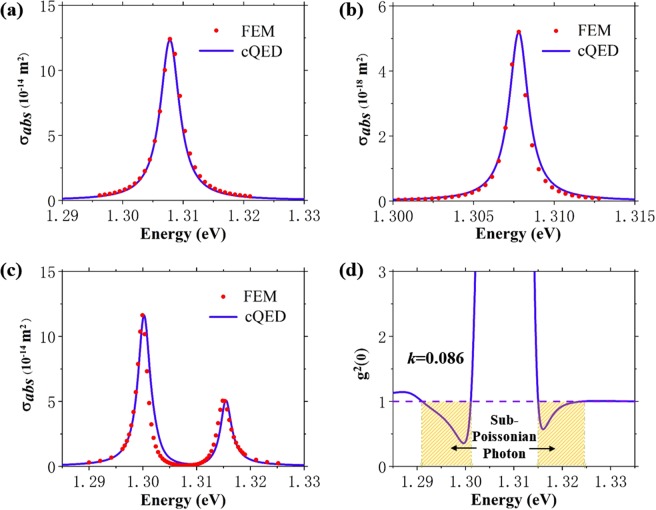
Calculated (FEM) and numerical (cQED) absorption cross section spectra of (**a**) bare cavity, (**b**) QD and (**c**) QD-MNPs hybrid system at *k* = 0.086. (**d**) Zero delay second-order autocorrelation function *g*^2^(0) as a function of incident wave ℏω. Shadow areas indicate the regions for generation of sub-Poissonian light.

Next, gain coefficient *k* = 0.086 is taken as an exemplary case to demonstrate the photon statistical properties in the proposed hybrid system. The process of obtaining quantum parameters can be divided into three steps: (1) Isolated cavity. Values of *ω*_*s*_, *κ*, and *d*_*s*_ are determined by fitting the single-peak spectra of cavity simulated by FEM and cQED as shown in Fig. 3(a), respectively. Within the framework of cQED, the physical quantities related to QD and coupling item in Hamiltonian are ignored. (2) Isolated QD. Similarly, the quantities related to isolated QD including *ω*_*q*_, *γ*_2_, *d*_*q*_ and ${\gamma }_{1}=\sqrt{{\varepsilon }_{d}}{d}_{q}^{2}{\omega }_{q}^{3}/{(3\pi {\varepsilon }_{0}\hslash c)}^{3}$ can be obtained by fitting single-peak spectra shown in Fig. 3(b). To ensure the strongest interaction, the resonance between QD and cavity is guaranteed in our simulation. (3) QD- MNPs coupling system: Fano resonance, caused by the interference between QD and LSPs, can be observed in Fig. 3(c). The value of *g* is given by fitting the spectra of the hybrid system. Besides, the occurrence of QD affects the electric field near the MNPs, and vice versa. Thus, values of *d*_*s*_ and *d*_*q*_ are re-determined for reasonable fitting result. Through the above three steps, the eight parameters for *k* = 0.086 are determined: *ℏω*_*s*_ = 1.3078 eV, *d*_*s*_ = 1780D, *ℏκ* = 3.8 meV, *ℏω*_*q*_ = 1.3078 eV, *d*_*q*_ = 430D, *ℏγ*_1_ = 66.8 μeV, *ℏγ*_2_ = 0.4 meV, *ℏg* = 7.6 meV. From cQED perspective, difference between eigen-frequencies of splitting peaks in Fig. 3(c) equals 2*g*. The difference between magnitudes of two splitting peaks only relates to *d*_*q*_, while *d*_*s*_ affects the magnitude of overall spectrum. The criterion *g*/*κ* = 2 quantitatively reflects the strong coupling between QD and cavity. When gain media is absent, *ℏκ* = 88 meV and *ℏg* = 7.6 meV indicate relatively weak coupling. The appropriate introduction of gain media obviously helps to achieve the strong coupling which is beneficial for sub-Poissonian photon statistics. On the other hand, the increase of QD dipole moment from 42D (*k* = 0) to 430D (*k* = 0.086) also results from gain media. Using above eight parameters, *g*^2^(0) of the hybrid system are evaluated within the framework of cQED, as shown in Fig. 3(d). If we assume *ω*_*q*_ = *ω*_*s*_ = *ω*_0_, the detuning between the incident wave and the cavity or QD is Δ = *ℏ*(*ω*_*l*_ − *ω*_0_). When *ℏω*_*l*_ = 1.3e V, *g*^2^(0) reaches the minimum value 0.356 and Δ = 7.8 meV = −1.03 *ℏg*. In JC model, the perfect photon blockade takes place at exactly Δ = ±*ℏg*, leading to ideal sub-Poissonian photon statistics (*g*^2^(0) = 0)^27^. However, the existence of *κ* causes an energy eigen-states broadening, making the photon blockade practically take place at farther position, such as Δ = −1.03 *ℏg* in this work. The gap between the eigen-states of the first excited-state and those of the second excited-state gets smaller along with the decrease of *g*/*κ*, leading to an increased probability of second excited-state occupation. As a consequence, photon blockade effect is weakened and *g*^2^(0) increases. Since *κ* cannot be completed eliminated, the proposed QD- MNPs hybrid system is not ideal, however, *g*^2^(0) = 0.356 is an acceptable result for the simplest JC model^41^. The criterion *g*^2^(0) < 1 reflects that the gain media assisted QD-MNPs hybrid system can generate sub-Poissonian light when the incident wave *ℏω*_*l*_ ranges from 1.291 eV to 1.301 eV as well as from about 1.315 eV to 1.325 eV, as shown in the shadow areas in Fig. 3(d).

**Figure 4:**
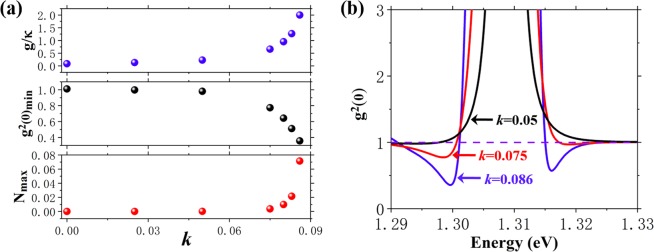
(**a**) Ratio of coupling strength to decay rate *g*/*κ*, the minimum zero delay second-order autocorrelation function *g*^2^(0)_*min*_ and the maximum number of intra-cavity photon in sub-Poissonian statistics *N*_*max*_ as a function of *k*. (**b**) Zero delay second-order autocorrelation function *g*^2^(0) as a function of incident wave *ℏ*ω at *k* = 0.05, *k* = 0.075 and *k* = 0.086.

The influences of gain coefficient *k* on performances of the proposed system is shown in Fig. 4(a), where only insufficient gain range to achieve optimum Q factor is under consideration. Mechanistically, the sub-Poissonian photon statistics is achieved by photon blockade, which requires a high value of *g*/*κ*. The proper presence of gain media is beneficial for boosting *g*/*κ*. The strong coupling (*g*/*κ* > 1/2) appears from *k* = 0.075, suggesting that *k* can play the regulatory role between weak and strong coupling regime. When *k* > 0.025, the corresponding minimum second-order autocorrelation function *g*^2^(0)_*min*_ keeps below 1, indicating that the hybrid system can generate sub-Poissonian light in such large range of *k*. *g*^2^(0)_*min*_ = 0.356 can be achieved at *k* = 0.086. The cases of *k* = 0.05, *k* = 0.075 and *k* = 0.086 are taken as examples to show how *g*^2^(0) changes with *k*, as demonstrated in Fig. 4(b). The Fock space is truncated at *F*_*cut*_ = 10 in cQED, which means the Hilbert matrix space is 10 × 10 dimensional. The value of *F*_*cut*_ is validated to provide consistent solution. In JC model, *F*_*cut*_ below 5 in the calculation is commonly adopted, thus *F*_*cut*_ = 10 is reasonably large for a QD-cavity coupling system. The higher quality factor achieved by the gain media (needing larger *F*_*cut*_ to fit in cQED) is not included in present work. Another aspect of our concern is the number of intra-cavity photon in sub-Poissonian statistics *N*, whose higher value is preferred. As *g*^2^(0) reaches the minimum value, *N* reaches the maximum *N*_*max*_ with the same incident wave energy. *N*_*max*_ = 0.07 appears at *k* = 0.086. Compared with *g*^2^(0)_*min*_ = 1.01 and *N*_*max*_ = 2.92 × 10^−5^ when gain media is absent, we learn that the appropriate *k* does not only help to generate sub-Poissonian light, but also increase the number of intra-cavity photons. It should be noted all decoherent photons produced by gain media are assumed depleted by the photon-plasmon coupling process. If excessive gain exists, the decoherence channel should be considered and might cause a compromise of the proposed scheme.

**Figure 5:**
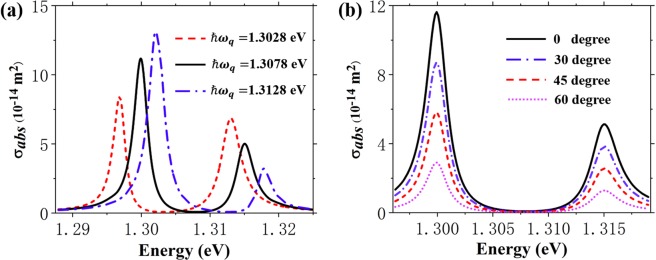
FEM calculated absorption cross section spectra of the hybrid system (**a**) with different *ℏω*_*q*_ at *k* = 0.086; (**b**) excited by incident wave with 0, 30, 45 and 60 degree polarization angle with respect to major axes of MNPs at *k* = 0.086.

Finally, the effects of non-ideal cases are considered in FEM simulations, including energy detuning and polarization mismatch. For energy detuning between QD and cavity, we take *k* = 0.086 as an example and calculate the absorption cross section spectra of the hybrid system with three values of *ℏω*_*q*_. As shown in Fig. 5(a), the solid curve represents no detuning case while the other two represent detuning *ℏ*(*ω*_*s*_ − *ω*_*q*_) = ±5 meV, respectively. Even if the detuning is greater than FWHM of the cavity (3.8 meV), the Fano resonance in spectrum induced by the strong coupling between QD and MNPs is not compromised. The spectra with detuning still can be fitted under the framework of cQED by further adjusting the quantum parameters. Our system shows a reasonable tolerance for detuning between QD and cavity. For polarization mismatch between the incident wave and cavity, the FEM simulated spectra of the coupling system excited by incident wave with 0, 30, 45 and 60 degree polarization angle (the angle between major axes of MNPs and the polarization of incident wave) are shown in Fig. 5(b). The major difference is the magnitude. Further fittings tell that the polarization mismatch between cavity orientation and incident wave has trivial effect on *g*^2^(0) and the value of *g*/*κ*, while it has significant impact on the intra-cavity photon number *N*, who drops from 0.07(0°) to 0.047(30°), 0.033(45°) and 0.008(60°). It suggests that the polarization mismatch between cavity orientation and incident wave could lead to a decline in driving field intensity, which can result in a substantial drop of *N* in theory.

## Conclusions

In summary, we have theoretically demonstrated a nanoscale gain-assisted QD-MNPs hybrid system generating sub-Poissonian light. The gain media enhances the quality factor of MNPs by the dynamic feedback between them. Under the assumption of complete depletion of gain-induced decoherent photons, proper choice of gain coefficient *k* leads the hybrid system falling in the strong coupling regime. The zero delay second-order autocorrelation function *g*^2^(0) = 0.356 is achieved, together with intra-cavity photon number *N* = 0.07. Moreover, the hybrid system is capable to maintain sub-Poissonian photon statistics in a large variation range of gain coefficient and tolerant to a certain degree of detuning between QD and cavity, benefiting the experimental realization.
